# Development of an intelligent motion controller and its application to the automation of a McBain-Bakr balance

**DOI:** 10.1155/S1463924693000252

**Published:** 1993

**Authors:** Jan Malczyk, Orhan Talu, Robert Megargle

**Affiliations:** 1 Department of Chemistry Cleveland State University Cleveland Ohio 44115 USA; 2 Department of Chemical Engineering Cleveland State University Cleveland Ohio 44115 USA

## Abstract

This paper presents a modular approach to motion control using a microconlroller-based stepper motor driver. As an application of the driver, an inexpensive and portable one-dimensional robot was built to automate an existing experimental setup for measurement of adsorption isotherms using the McBain-Bakr technique. Automatic recording of position versus time yields data for the sludy of adsorption/desorption dynamics.


Journal of Automatic Chemistry, Vol. 15, No. 5 (September-October 1993), pp. 183-187

### Development of an intelligent motion controller and its application to the automation of a McBain-Bakr balance

**Jan Malczyk, Orhan Talu* and Robert Megargle**
*Department of Chemistry, Cleveland State University, Cleveland, Ohio 44115, USA*

*This paper presents a modular approach to motion control using a microcontroller-based stepper motor driver. As an application of the driver, an inexpensive and portable one-dimensional robot was built to automate an existing experimental setup for measurement of adsorption isotherms using the McBain-Bakr technique. Automatic recording of position versus time yields data for the study of adsorption/desorption dynamics.*

### Introduction

Stepper motors are increasingly popular in many cost-effective designs of laboratory automation: they are ideal for position-control tasks and are easily interfaced to digital equipment. Their limitations, however, force careful consideration of the driver design when available commercial controllers are not suitable for a particular project. This paper describes the development of a flexible motion control system for a multi-axis laboratory robot, whose number of degrees of freedom would be changeable. It was important that this be accomplished without much modification of software and hardware of a host controller. As an intermediate goal, a one-dimensional robot was developed.

It was decided that each stepper motor should be controlled by a separate microcontroller, which is basically a small microcomputer in a single chip. This was attractive for several reasons:

(1) Each microcontroller takes over the chores of sequence switching, speed control, reaching final position, limit switch sensing, and lowering power to the stepper motor when it is not moving.

(2) Serial communication allows for long distances between such a driver and the master controller computer. This method of communication reduces the number of wires, and the small size of the driver allows mounting close to the motor.

(3) Driving parameters can be individually optimized for different types of stepper motors and different applications.

(4) The intelligent controller module can be made with error detection and handling capabilities.

Using a devised set of commands, any task can be accomplished by sending a particular instruction code. If that task cannot be performed, the host is notified about the problem by a returned error code. The hardware of the module can be changed, and new commands can be added without much modification of the host software.

### Description of the controller

The Zilog Z8613 (Protopack) single-chip microcomputer [1] was selected for prototyping the controller design (figure 1) to drive a two-phase stepper motor in unipolar mode. The first four lines ($P0_0$–$P0_3$) of Port 0 (P0) are buffered (B), optically isolated (OI1), and used to provide ON/OFF signals to HEXFET transistors (T) which drive the coils of a stepper motor (L1–L4). The state of each line is indicated by an LED (D). Line $P0_4$ is the master enable/disable for the $P0_{0-3}$ lines and puts the four buffer outputs to high impedance state upon POWER ON, or RESET, to avoid any unwanted stepping. The $P0_5$ line is buffered (B), optically isolated (OI2), and used to provide full or half power to the motor. Half power is used when the motor is stationary. This solves the problem of heat dissipation that would be generated by the motor if full power was used all the time.

The $P0_{6-7}$ and $P2_4$ lines control operation of the HP2016 counter chip (EC). The chip performs the quadrature decoder function of the two squares waves generated by an optical encoder (OE), which is mechanically linked to the stepper motor. The current position of the motor shaft can be monitored by reading the 16-bit counter value via lines $P1_{0-7}$ of Port 1. Lines $P2_{0-1}$ monitor the state of the limit switches (SW1–SW2). The controller is programmed to immediately stop the motor if the appropriate limit switch is closed.

The $P2_{2-3}$ lines are used for baud rate selection. During initialization, the microcontroller reads the state of these inputs, which are determined by jumpers (J1–J2), and selects a rate: 2400, 4800, 9600 or 19200 Baud. The $P3_0$ and $P3_7$ lines are designed in the Z8613 to be used for serial-in and serial-out communication. They are buffered (B) to TTL levels and may be transformed to RS-232 standard voltage with a separate circuit, such as MAX232. They can then be directly interfaced to a computer equipped with a standard serial port. The RESET line can be brought active low, either by an on-board push button (PB) switch or by an external digital line (ERL). A few I/O lines ($P2_{5-7}$) and interrupt lines ($P3_{1-3}$) are left available for additional applications.

The 5V power for the controller logic is obtained from a 7V unregulated source using a LM340T regulator (RG). The 7V source is used to half power to the motor, while a 12V source delivers full power. Both power sources

* *Department of Chemical Engineering, Cleveland State University.*

0142-0453/93 $10.00 © 1993 Taylor & Francis Ltd.

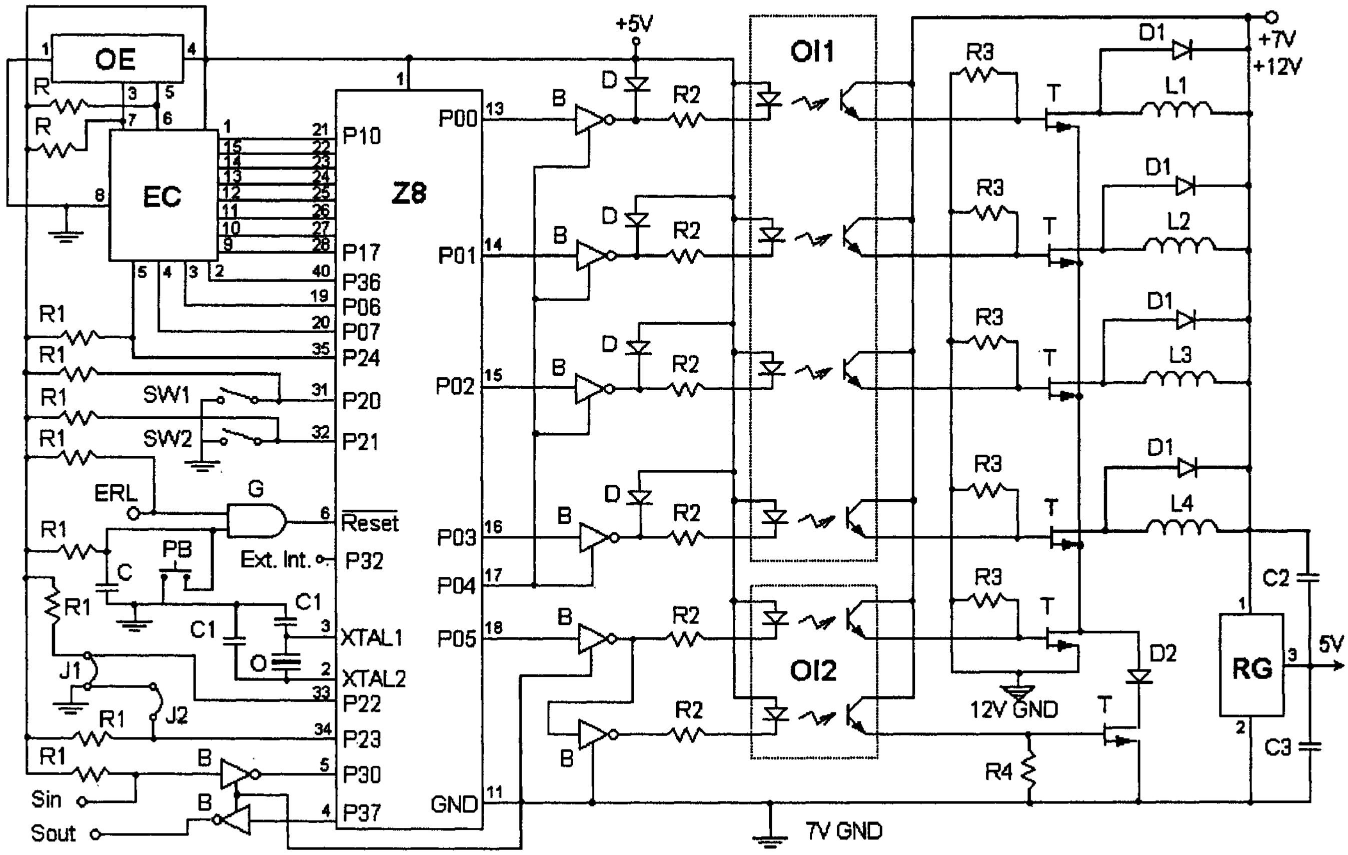

*Figure 1. Schematic of the intelligent stepper motor controller.*

| | | |
|---|---|---|
| B—1/8 of HCT240 | ERL—External reset line | R1—4.7 kΩ |
| C—2.2 μF | G—1/4 of LS08 | R2—1 kΩ |
| C1—6.8 pF | J1 and JS—Jumpers | R3 and R4—4.7 kΩ |
| C2—0.22 μF | O—7.3728 MHz oscillator | RG—LM340T |
| C3—0.1 μF | OE—Optical encoder (E2-400-250-PTE) | Sin—Serial input |
| D—4 mA LED | OI1—PS2004 | Sout—Serial output |
| D1—1N4148 | OI2—PS2002 | SW1 and SW2—Limit switches |
| D3—1N5403 | PB—Push button | T—IRFZ22 |
| EC—HCTL2016 | R—2.7 kΩ | Z8—Z8613 |

do not need to be well regulated, and power supplies can be made very simple at low cost. Because motor coils can produce high voltage spikes during switching, some of the more complex regulated power supplies do not work well. The two power sources are used in an unusual configuration. The positive outputs are connected and two separate grounds are used. This allows the HEXFETs (T) to be operated without heat sinks, even at currents exceeding 1A per phase. This substantially contributes to the compact size of the controller board (6 × 9.5 × 2.5 cm).

The controller software resides in the internal 4k ROM memory of the Z8613. The software was designed to allow for continuous communication with the host. After initialization, a READY state is signalled by sending out a specific code and the program enters the main dispatch loop to wait for a task to perform. The interrupt driven routine for serial communication determines the validity of any received command opcode and then determines the number of additional bytes (data) to be received. For reasons of reliability, every received bytes is echoed back. Upon successful communication from the host, an appropriate command handler is employed, a BUSY flag is set, and the command is executed. After execution, the BUSY flag is cleared, the READY code is sent to the host, and the program returns to the main loop.

If an attempt is made to send another command before finishing the current one, a BUSY state code is usually returned to the host and and the attempted command is discarded. However, there are some commands and error conditions that require immediate attention by the microcontroller. Therefore, some commands are acted on immediately, such as stopping the current action or reporting the current counter value of the optical encoder.

The commands and error codes form an easy to learn language for operating the controller. The FIND_SPAN command determines the allowed range of movement between limit switches in encoder counter units. One full shaft rotation is equivalent to 1600 counts, 200 full steps, or 400 half steps. A motor move can be performed by the ROTATE_RIGHT_N and ROTATE_LEFT_N commands, where N is the number of motor half steps; or by the REACH_POSITION_X command, where X is the desired final position in encoder counts. FIND_HOME brings the motor to a well-defined position, usually determined by a limit switch. The encoder counter output is reset to zero when the home position is reached as part of the command. RESET_COUNTER clears the 16-bit encoder counter (HCTL2016), and REPORT_COUNTER gives the current position of the motor shaft. LOAD_REGISTER allows the user to change the default parameters, for example maximum speed, by changing the contents of a given Z8613 register. In particular, a different entry of an interrupt handling routine can be used depending on the current contents of another dedicated register. The reverse operation, READ_REGISTER, allows the host to investigate any of the general-purpose and some of the control registers. Thus, detailed motion control of a stepper motor consists of a few simple commands.

### Automation of McBain-Bakr technique

The McBain-Bakr technique is used for measuring adsorption isotherms and is of particular value in comparative studies [2]. It still finds its widespread use in the industrial environment because of simplicity of design, ease of use, reliability of data, and low cost. A set of five balances with a vacuum and vapour feed system was installed in the Advanced Manufacturing Center (AMC) of Cleveland State University for measurements of adsorption isotherms (see figure 2). Each balance consists of a large quartz tube connected to a vacuum and feed valve. Inside each tube there is a helical quartz spring to which a small sample holder is attached. The spring elongates as the sample mass increases due to adsorption of gaseous species. From the spring constant and the elongation, one can easily determine the amount of species adsorbed.

A cathetometer is often used for determination of the spring elongation. This method of collecting data is especially inadequate for dynamic studies of fast systems, for example adsorption of benzene vapours on carbon samples. Thus, the objective of the first stage of computerization was to automatically scan and record the position of a reference point near the sample using a simple robot to determine elongation versus time. Since the experiments are mainly conducted at ambient temperatures, an automatic scanning and data recording system could be designed easily.

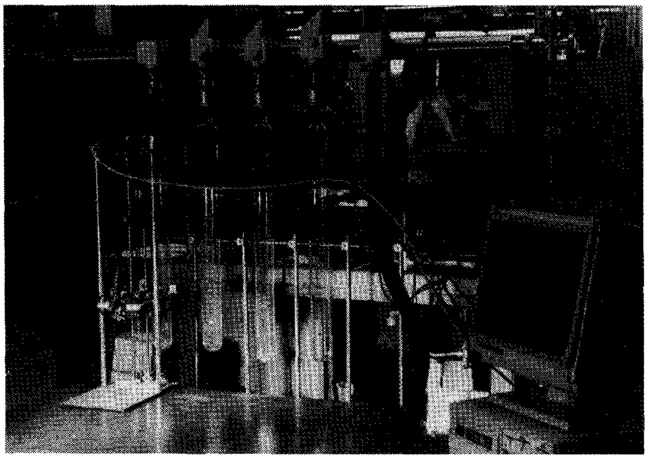

*Figure 2. View of the experimental set-up and the robot.*

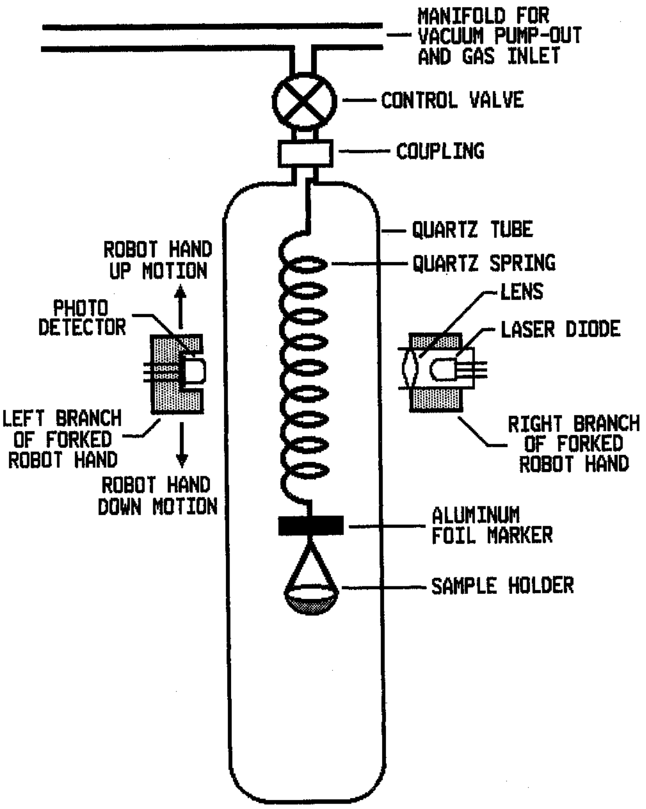

*Figure 3. Cross-section of the McBain-Bakr apparatus with position-sensing hand. Note on scale: the fingers of the robot hand are actually much smaller compared to the quartz tube than is shown in this figure.*

An opaque object, made of aluminium foil, was inserted between the quartz spring and the sample holder (see figure 3). To account for spring twisting during elongation a 'Z'-shaped object was chosen so that it blocks the light beam at all rotational positions. Aluminium foil was used to minimize weight (65 mg). The current position of the sample is determined when a laser beam is blocked by the foil. The laser light is produced by a 670 nm, 5 mW laser diode and collimated by a miniature lens. The diode and lens are housed in a small aluminium block mounted on the right-hand side of the robot arm. On the left-hand side of the fork-like arm, there is a light detector with logic output. Focusing and beam alignment are easily performed by appropriate adjustment of set screws.

Directing the light beam transversely through the quartz tube centre is accomplished by setting the entire robot arm unit properly on the bench. A small size for the optic system was achieved by placing the electronic circuitry on the top of the robot, with connecting wires to the diode and detector. A simple laser diode driver is used to supply constant current to the diode. The output of the detector is digitally filtered using a technique similar to that adopted for conditioning the signal from an optical encoder. The filter circuit changes its output when a light level change at its input is stable for three clock periods.

The user can regulate the clock period to optimize performance. The output of the filter circuit is connected to the pin $P3_2$ of the Z8613, which is an external interrupt line. An interrupt request is generated by a negative edge signal on $P3_2$ whenever the light beam is blocked.

Motion control is provided for the arm movement in a vertical direction. The maximum arm stroke, as measured by the cathetometer, is 476.2 mm. This distance corresponds to 15266 encoder counts, giving a theoretical resolution of 0.0312 mm. The stroke range can be changed by moving the limit switches to any desired location. The time between two successive scans is software controlled and ranges from about 3 s up to any value. The robot contains all electronic circuitry, with power supply, and can be easily moved to acquire data from any of the balances. An IBM PC is used to control the robot.

The software for data acquisition is written in the Turbo C++ language by Borland International, Inc. The operational codes of the commands operating the intelligent controller were given self-understandable names. The names serve as parameters in a C-program function that was written to control the robot. The function is basically a software interface between the high level language of the host and the Z8613 software. Additional parameters of the C-program function may contain data pertinent to some commands. The return value of the function carries information about execution status. Thus, the programmer perceives the problem of motion control as a function with up to three parameters and a list of a few easy-to-memorize commands. When more than one stepper motor is to be controlled, an additional parameter specifying a given motor (serial port address) will be required in this function.

The data acquisition software operates in the following way. During initialization, a new serial interrupt handler is substituted for the DOS version and the user selected serial port is initialized. A motor calibration step from the main menu is usually performed first to find the current range and allow the experimenter to check the beam path across the tube. Next, using menu options, some parameters like rate of data acquisition, can be selected. Alternately, the user can start an experiment with default parameters.

Upon start, the host commands the robot to FIND_HOME. The Z8613 moves the arm up until the upper limit switch is closed, retreats back 3 mm, and very slowly closes the switch again. When the switch is closed the counter for the encoder on the motor is reset. This initializes the measurement system. Before the ROTATE_LEFT_N command is given to the Z8613 to start a scan, two LOAD_REGISTER commands are issued by the host to modify default contents of two dedicated registers. The contents of one of the Z8613 registers determines the maximum scanning speed. The other register determines the mode of execution of the interrupt service routine which responds to an interrupt generated when the light beam is blocked during scanning. The interrupt service routine, when enabled, captures the position given by the counter after an interrupt has happened and immediately informs the host computer about this event so that the PC system time can also be recorded. Also, the interrupt handling routine prepares the motor to slow down and stop. It will not process any additional interrupts until the host reloads the dedicated register with an appropriate value; this value is automatically reset upon return from the interrupt service routine.

The captured position is read from the Z8613 registers by the PC using two READ_REGISTER commands. It is placed in a data buffer along with the current time. The arm is brought up a preset distance from the stopped position at a preselected speed with the ROTATE_RIGHT_N command. The robot waits for specific instructions to perform the next scan. All subsequent scans begin from this new starting position. They are initiated by the host at time intervals specified by the user at the beginning of the experiment, or from default values. The data acquisition can be stopped at any time and restarted again without any data loss. If the number of collected data points reaches a preselected buffer size, the data are automatically appended to a disk file opened for this experiment. All buffer results are moved to this disk file when this experiment is ended. The data saved in the disk file are analysed separately by other software.

### Results and discussion

A fixed opaque obstacle was used to test the repeatability of the position determination. The upper graph of figure 4 shows the result of 246 readings and the horizontal line represents the average value 8939 in counter units. The data set has a standard deviation $\sigma = 3$. Since the first derivative is used for diffusivity calculations from the time dependent adsorption data, the experimental data needs to be smoothed. The lower graph in figure 4 shows the result of smoothing by fitting the points with a least squares polynomial of third degree (it is drawn to scale, but shifted down 12 units for clarity). The mean value of the smoothed data set is also 8939 counts, but the standard deviation and the difference between maximum and minimum value are decreased threefold. The main contribution to the noise comes from the industrial environment of the AMC. Also, the stepper motor vibrations contribute

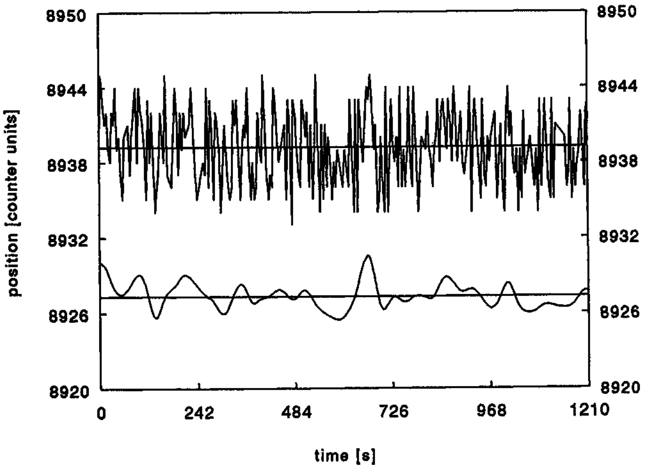

*Figure 4. Results of testing the reproducibility of 246 fixed-position readings. The upper part shows experimental data, and the lower curve is shifted down for clarity and shows the smoothed data.*

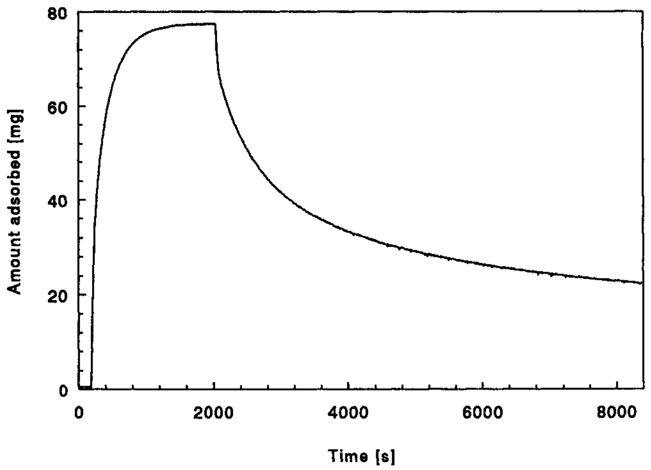

*Figure 5. Experimental data of adsorption/desorption of benzene vapour on a carbon sample versus time.*

to the noise. Improving the mechanical design and rigidity of the prototype robot, as well as installing a stepper motor with a low noise level, should diminish the noise from the instrument itself. Assuming random fluctuations in the position readings and accepting 99·9% confidence level for the smoothed data set, the product 3·29*$\sigma$ yields $\pm$0·1 mm error of a single measurement. This figure is no worse than the usual error obtained using a cathetometer, where the optical determination of the same spot from a distance of about 1 m contributes most of the uncertainty of the position reading.

The robot was then used for acquisition of data. A carbon sample (Type V6, Mesh 6 × 12, Lot# 3453 from Barnebey & Sutcliffe Corp.) of mass 195.4 mg was activated for 18 h at 246°C in the McBain-Bakr apparatus. A continuously working rotary pump was able to maintain a pressure below 0.3 Torr. After activation, the vacuum valve was closed and the temperature of the system equilibrated to the ambient temperature of 25°C. Before admitting benzene vapours to the sample, automatic recording was started. Figure 5 presents unsmoothed experimental data obtained by using the robot. The flat part at the beginning shows the stable weight prior to introduction of benzene vapour. The ascending part of the curve shows adsorption of benzene vapour on the carbon sample at an equilibrium pressure of 2.4 Torr. The descending part of the curve shows the slower desorption process which took place after opening the vacuum valve and continuously pumping out the benzene vapour. Since the spring constant is 1 mm/1 mg and benzene vapour adsorbs so easily on carbon, the spring elongation is quite large and reaches 100 mm at higher pressure.

Initial positions, at equilibrium, and at the end of the experiment, were measured independently using the cathetometer. Very good agreement with the data captured by the robot was achieved. The cathetometer readings at initial, at equilibrium, and at final positions were 678·60, 601·75, and 656·60 mm—giving total elongations of 76·85 mm for adsorption and 54·85 mm for desorption processes. The total elongations determined by the robot were 76·97 and 54·90 mm, respectively. The steep parts of the experimental curve at the beginning of adsorption and desorption occurred too fast to use the cathetometer for data acquisition. Measurement of this time dependency of adsorption, which is related to diffusivity, is the novel application of robotics to McBain-Bakr balance.

### Conclusions

A modular approach to motion control makes automation easier. Combinations of basic modules can be used to implement laboratory automation in a stepwise manner. The automatic recording system described in this paper provides data for study of adsorption/desorption dynamics at a given vapour pressure and temperature. Controlling the vapour feed valve to provide a desired vapour pressure would be the next step towards more advanced automation of the McBain-Bakr apparatus.

### References

1. Z8 Microcontrollers, Technical Manual (Zilog, 1991).
2. Young, D. M. and Crowell, A. D., *Physical Adsorption of Gases* (Butterworth & Co. (Publishers) Ltd, 1962).

